# Sales data as a measure of antibiotics usage: Concepts, examples and discussion of influencing factors

**DOI:** 10.1002/vms3.205

**Published:** 2019-10-21

**Authors:** Roswitha Merle, Borris Meyer‐Kühling

**Affiliations:** ^1^ Institute for Veterinary Epidemiology and Biostatistics Freie Universität Berlin Berlin Germany; ^2^ PHW‐Zentrallabor Visbek Germany

**Keywords:** antimicrobial consumption, cephalosporins, farm animals, PCU, quinolones, technical units

## Abstract

Monitoring and surveillance of antimicrobial usage in animals is a public health concern and different methods are currently discussed widely in public, science and politics. The objective of the paper is to present the available methods of monitoring and to discuss possible differences in the assessment of the antibiotics treatment. Sales data are expressed as the average amount of substance per animal or kg live weight (e.g. population‐corrected unit, PCU). The number of Defined Daily Doses (nDDDvet) is calculated by extrapolating sales data with average animal weights and defined drug doses to a number of treatments theoretically applied to animals. In contrast, the number of Used Daily Doses (nUDDvet) displays the actual number of treatments which have been applied. As sales data are relatively easily obtained, they are frequently used. However, their results are influenced by the composition of the population and by the dose of the substances. As both may vary strongly between countries, direct comparison of sales data between countries may be misleading. The concept of analysing sales data is shown by exemplarily using the methods in the ESVAC report 2015. The presentation of usage data in terms of nDDDvet or of nUDDvet increases the comparability of the data from different countries or time periods. Furthermore, fluoroquinolones and third‐/fourth‐generation cephalosporins which, among other substances, bare a potential risk for human health, are used at low doses. Hence, their use contributes to a sales reduction while contrasting the guidelines of prudent use. nDDDvet or nUDDvet have the ability to better reflect the treatment frequency and thus to better link antibiotics use to public health concerns. Quantification of antibiotics should assist to focus on prudent use of antimicrobials to reduce the burden of resistant bacteria and, thus, enhance public health, animal health and animal welfare.

## Graphical abstract

Usage of antibiotics in livestock is often expressed in terms of usage in mg/kg live weight. This is influenced by the percentage of live weight that is related to pigs and poultry as well as by the choice of the active substance. In consequence, the important influence factors such as animal health and welfare conditions may remain undetected.

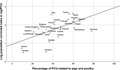

## 1 INTRODUCTION

The documentation of the antibiotic consumption both of humans and animals is an urgent global one health need (World Health Organization, 2015). For this, sales data are commonly used to display the usage of antimicrobials in animals. These data are derived from marketing authorization holders, wholesalers or feed mills and are relatively easy to obtain. An average consumption per animal can be calculated by dividing the sum of sold active substances by any population unit, for example, number of animals or kg biomass produced. Many countries report sales data and related units of measurement such as mg/biomass regularly (Bager et al., 2015; Belgian Veterinary Surveillance of Antibacterial Consumption National consumption report, ; The Public Health Agency of Sweden & National Veterinary Institute, 2014; Veterinary Medicines Directorate, 2012). Sales data are also used in projects and reported in the respective publications (Bondt, Jensen, Puister‐Jansen, & van Geijlswijk, 2013; Carmo et al., 2017; Menéndez González, Steiner, Gassner, & Regula, 2010).

The project European Surveillance of Veterinary Antimicrobial Consumption (ESVAC) of the European Medicines Agency (EMA) was established in 2009 and has been reporting sales data in European countries since. Countries take part voluntarily and the most recent report from 2016 states a total of 30 countries (27 EU member states, 2 EEA countries as well as Switzerland) providing data (European Medicines Agency und European Surveillance of Veterinary Antimicrobial Consumption, 2018); Grave et al., 2014; . Results are reported in terms of population‐corrected sales in mg of substance per population‐corrected unit (PCU) (European Medicines Agency, 2017; Grave et al., 2014).

Although easily obtained, the presentation of antibiotics usage in terms of sales data is prone to being influenced by factors of several types. Both the numerators, that is, the amount of substances, as well as the denominator, that is, the population at risk, underlie various factors, which may strongly influence the outcome. Consequently, data from different populations, for example, countries, or time periods cannot be compared directly and may lead to misinterpretation of the results (Bondt et al., 2013).

Collineau et al. (2017) recently published a detailed overview and discussion regarding the indicators for quantification of antimicrobial usage and their applications. They point out that national‐level data are useful for certain study objectives such as monitoring trends over time, but only if comparability of the populations is given. Lekagul et al. (2018) as well as Werner, McEwen, and Kreienbrock (2018) also reviewed the diversity of methods for quantification.

The purpose of the present publication is to explain the pitfalls and consequences of using sales data for inter‐country comparison, that is, the challenges regarding the composition of animal husbandry forms of the countries, the different dosing regimens of substances and the different impact of substances regarding public health. To demonstrate these restrictions, the concept of analysing sales data is shown briefly by exemplarily using the methods in the ESVAC report 2015 (European Medicines Agency, 2017).

Additionally, alternatives to sales data such as the quantification of defined or actual treatments are presented and discussed.

## 2 MATERIALS AND METHODS

Methods are described in general, details refer to the ESVAC report 2015 as example (European Medicines Agency, 2017), which is known to be the most comprehensive system on antibiotic sales data documentation in the world and has been evaluated by a large group of experts from the European countries.

### 2.1 Population at risk

The definition of the population at risk of being treated is a crucial variable for which several different calculation approaches are available (Collineau et al., 2017). One concept is to identify the biomass or live weight at risk of being treated by multiplying the number of produced or live animals with the respective expected body weight at typical treatment age. The expected body weight differs between animal species, age or production groups. Alternatively, the number of animals at risk of being treated can be used.

ESVAC uses the PCU (=1 kg live weight) and refers to Montforts (2006) regarding animal weights (Table 1) (European Medicines Agency, 2009; Montforts, 2006). Other publications report different animal weights (e.g. Bager et al., 2015; Jensen, Jacobsen, & Bager, 2004; SDa expert panel, 2015; The Public Health Agency of Sweden & National Veterinary Institute, 2014).

**Table 1:** Average animal weights at typical age of treatment (European Medicines Agency, 2009; Montforts, 2006)

Animal category	Weight in kg
Slaughtered cows, bulls or bullocks; dairy cows	425
Slaughtered heifers	200
Slaughtered calves and young cattle; feeding cattle	140
Living sows	240
Slaughtered pigs	65
Fattening pigs	25
Living sheep	70
Slaughtered sheep and goat; fattening sheep and goat	20
Turkey	6.5
Broilers	1
Horses	400
Rabbits	1.4
Fish	Biomass slaughtered weight

ESVAC’s PCU is calculated as the sum of all animals multiplied with their respective body weight. The number of animals consists of slaughtered plus live animals (for dairy cattle, sows, horses and sheep).

### 2.2 Sales data

The amount of antibiotics sold is expressed as amount of antimicrobial substances in tonnes and is usually completed by information on the substance and its pharmaceutical form. The countries submit the number of sold packages per product and package size to ESVAC. The countries also provide information on name and concentration of the active substance(s). ESVAC calculates the sum of all active substances sold per country in tonnes.

ESVAC reports the results as mg/PCU, that is, the relation of the overall amount of substances to the overall PCU. In the ESVAC report 2015, the results ranged from 2.9 to 434.2 mg/PCU per country (European Medicines Agency, 2017).

### 2.3 Quantification of defined treatments

The frequency of treatments is described by means of technical units to achieve comparable values. Definitions of this measure can be found in the literature under different similar notations, for example, DDD, ADD or DDDA. ESVAC’s term DDDvet is used throughout this publication (European Medicines Agency, 2016, 2015).

The Defined Daily Dose DDD is the dose of a substance applied to an animal in one day when treated with one defined dose. The defined dose is determined for each substance, each animal species and each administration route separately. Combination drugs or long‐acting compounds also require separate doses.

Several studies report different values of DDDs, rendering their results not directly comparable (Bondt et al., 2013; Callens et al., 2012; Grave, Kaldhusdal, Kruse, Harr, & Fevang; Flatlandsmo, Knut, 2004; Persoons et al., 2012; Postma et al., 2015). In 2016, EMA has published doses that are used for the examples shown in this article (European Medicines Agency, 2016, 2015).

The number of DDDvet (nDDDvet) is calculated by dividing the amount of the respective substance used by its DDD. Division of the nDDDvet by the population at risk (as described above, also see Collineau et al., 2017) results in an average nDDDvet per population unit, for example, per animal or per PCU. It can be displayed separately for each substance and/or administration route or as an overall result.

Formulas 1 and 2 are used to calculate the examples displayed below.

(1) $$\text{nDDDvet}=\frac{\text{amount of substance}}{\text{Defined Daily Dose}}$$

(2) $$\text{nDDDvet}/\text{PCU}=\frac{\text{nDDDvet}}{\text{number of animals}\;\times \;\text{average animal weight}}$$

### 2.4 Quantification of actual treatments

UDDvet is the used daily dose that is applied to an animal on one day. The number of UDDvet (nUDDvet) is calculated by multiplying the number of treated animals with the number of treatment days and the number of different substances applied. The sum of all nUDDvet during a certain time period is divided by the population at risk and results in the nUDDvet per animal or PCU (Chauvin, Beloeil, Orand, Sanders, & Madec, 2002; Menéndez et al., 2010; Obritzhauser et al., 2016; Persoons et al., 2012; Timmerman et al., 2006; Trauffler, Griesbacher, Fuchs, & Kofer, 2014; van Rennings et al., 2015).

The following formulas 3 and 4 are suitable for these means of calculation.

(3) nUDDvet=number of treated animals×treatment days×number of substances

(4) $$\text{nUDDvet}/\text{PCU}=\frac{\text{nUDDvet}}{\text{number of animals}\;\times \;\text{average animal weight}}$$

Inaccuracies occur when long‐acting compounds are used that are effective for more than 24 hr. Correction may be performed by replacing treatment duration by duration of effectiveness typical of this substance, but currently no internationally accepted solutions for this problem exist.

### 2.5 Examples

To illustrate differences in the results depending on the calculation method used, the treatment of respiratory diseases in broilers is given as an example. The mg/PCU of the specific treatments is calculated following formulas 5 and 6.

(5) $$\begin{array}{cc} \text{amount substance}\; & =\;\text{number of animals}\times \text{animal weight}\\ & \;\times \text{dose}\times \text{treatment}\;\text{duration} \end{array}$$

(6) $$\begin{array}{cc} \text{amount}\;\text{substance}/\text{PCU} & =\frac{\text{amount substance}}{\text{number}\;\text{of}\;\text{animals}\;\times \;\text{animal weight}}\\ & =\text{dose}\;\times \;\text{treatment}\;\text{duration} \end{array}$$

### 2.6 Statistical analyses

Statistical analyses were carried out with IBM SPSS Statistics version 24. Published data of the ESVAC report 2015 were used to carry out the following analyses: the variable ‘animal density in PCU/km^2’^ was calculated by dividing the overall number of PCU (European Medicines Agency, 2017) by the area of the respective country (://www.wikipedia.com) in square kilometres. Additionally, the variable ‘percentage of the overall PCU related to pigs and poultry (intensive animal husbandry species)’ was calculated.

Variables were checked for normality by Shapiro–Wilk test as well as visually. ‘Population‐corrected sales in mg/PCU’ was transformed to logarithmic values (to the base of 10) to achieve normal distribution.

Pearson's correlation coefficient was calculated to display the correlation between the percentage of overall PCU related to pigs and poultry and the population‐corrected sales in mg/PCU (log). To investigate the influence of the percentage of PCU related to pigs and poultry as well as the animal density in PCU/km^2^ on the population‐corrected sales in mg/PCU (log), a multivariable linear regression model was adjusted. Values of the independent variables were centred. The animal density did not show a linear relationship to log mg/PCU but its cubic transformation x_cubic = x * x^2^ *x^3^ fitted well to the data. Thus, the variables ‘animal density’, ‘(animal density)^2’^ and ‘(animal density)^3’^ were included in the linear regression model. The impact of the model was evaluated by the adjusted R squared. Normality and homoscedasticity of residuals were checked visually.

## 3 RESULTS

**Figure 1:**
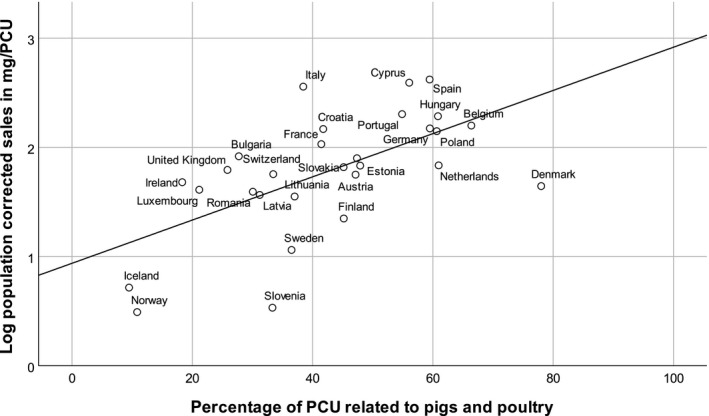
Relationship between the percentage of PCU related to pigs and poultry and the antibiotics usage in log mg/PCU following the ESVAC report 2015 (European Medicines Agency, 2017)

The percentage of PCU related to pigs and poultry (intensive animal husbandry species) as reported in the ESVAC report 2015 ranged from 9% to 79% (European Medicines Agency, 2017) and showed moderate correlation to the log population‐corrected sales in mg/PCU (Pearson's *r* = .586, Figure 1).

Since a high animal density (PCU/km^2^) correlated with a high percentage of intensive animal husbandry species (Pearson's *r* = .479), we investigated whether the percentage of intensive animal husbandry species or the animal density had significant effects on the overall population‐corrected sales in log mg/PCU. The multivariable regression analysis (*p* < .001) demonstrated that the percentage of PCU related to intensive animal husbandry species (*p* < .001) as well as the cubically transformed animal density (*p* = .002 for animal density, *p* = .003 for (animal density)^2^, *p* = .010 for (animal density)^3^) had statistically significant effects on log mg/PCU (Table 2, Table 3).

**Table 2:** ANOVA table of linear regression analysis, log mg/PCU as dependent variable, percentage of PCU related to pigs and poultry as well as animal density as independent variables

	Sum of squares	Degrees of freedom	Mean squares	*F* value	*p* value
Regression	4.042	4	1.010	8.979	<.001
Residuals	2.813	25	0.113		
Total	6.855	29			

**Table 3:** Coefficients of linear regression analysis, log mg/PCU as dependent variable, percentage of PCU related to pigs and poultry as well as animal density (cubic transformation) as independent variables

	Regression coefficients b	*t* statistic	*p* value	95% Confidence interval	
				Lower limit	Upper limit
Constant	1.784	29.129	<.0001	1.658	1.910
Percentage of PCU related to pigs and poultry^a^	0.020	4.675	<.001	0.011	0.028
Animal density in PCU/km^2^ a	0.076	3.455	.002	0.031	0.121
(Animal density in PCU/km^2^)^2^ a	−0.002	−3.243	.003	−0.004	−0.001
(Animal density in PCU/km^2^)^3^ a	0.00002	2.787	.010	0.000	0.000

^a^ Variables were centred. Constant corresponds to 41.4% pigs and poultry at 15.97 PCU/km^2^, 577.22 (PCU/km^2^)^2^ and 31,937 (PCU/km^2^)^3^.

The regression coefficient of.020 is defined as the population‐corrected sales increased by 0.020 log mg/PCU when the percentage of PCU related to pigs and poultry increased by 1 percentage point. The adjusted *R* squared of .524 indicated that more than half of the variation of log mg/PCU could be explained by the variables in the model.

### 3.1 Relationship between sales, nDDDvet and nUDDvet

The relationship between sales, nDDDvet and nUDDvet, is displayed by the following example: respiratory diseases in broilers can be treated with different substances. As example, 100 broilers of 800 g live weight each must be treated either with tylosin (macrolide) or with a combination of lincomycin (lincosamides) and spectinomycin (aminoglycosides).

The veterinarian decides to apply tylosin with a dosage of 100 mg/kg via drinking water for 3 days (recommendation: 20–100 mg/kg for 3–5 days (Löscher, Richter, & Potschka, 2014)). Alternatively, the veterinarian decides for a treatment of the combination preparation with 17 mg/kg lincomycin and 34 mg/kg spectinomycin for 4 days (recommendation: 17–25 mg/kg lincomycin and 34–50 mg/kg spectinomycin for 4–7 days (Löscher et al., 2014)). The amount of substance per PCU is calculated following formula 6.

Treatment with tylosin results in.

100mg/kg·3days=300mg/PCU,

while treatment with the combination preparation results in.

$$\left(17+34\;\text{mg}/\text{kg}\right)\;\cdot \;4\;\text{days}\;=\;204\;\text{mg}/\text{PCU}\;\left(\text{Table}\;4\right).$$

The nDDDvet is calculated as introduced above (formulas 1 and 2). It needs to be considered that the average animal weight = PCU =1 kg is used in these calculations instead of the actual animal weight = 0.8 kg. Based on the calculated usage data, treatment with tylosin results in 2.96 nDDDvet per PCU (Table 4) while treatment with the combination of lincomycin and spectinomycin results in 5.34 nDDDvet per PCU.

**Table 4:** Example calculation of the amount of substances used in mg/PCU, in nDDDvet/PCU as well as in nUDDA/PCU, when 100 broilers are treated with different substances (PCU = 1 kg)

Substance	Number of animals	Actual weight (kg)	Treatment duration (days)	Dose (mg/kg body weight)	Amount substance (mg)	mg/PCU	Average weight (kg) = PCU	Defined dose mg/kg	nDDDvet	nDDDvet/PCU	Number of substances	nUDDA	nUDDA/PCU
Tylosin	100	0.8	3	100	24,000	300	1	81	296.3	2.96	1	300	3
Lincomycin	100	0.8	4	17	5,440	68	1	22	247.3	2.47	1	400	4
Spectinomycin	100	0.8	4	34	10,880	136	1	38	286.3	2.86	1	400	4
Combination Lincomycin & Spectinomycin	100	0.8	4	51	16,320	204	1		533.6	5.34	2	800	8

The calculation of the number of UDDvet is carried out following formulas 3 and 4.

Therefore, the treatment of the 100 broilers results in 3.0 nUDDvet/PCU for tylosin and 8.0 nUDDvet/PCU for the combination preparation (Table 4).

## 4 DISCUSSION

### 4.1 Sales data

Sales data are usually reported on national level and thus summarize the results over all animal species and substances included. Direct comparison of data from different populations, regions or time periods may be influenced, among others, by two factors: the composition of the population and the doses of the substances.

### 4.2 Composition of animal populations

If countries or time periods are compared, the composition of the population usually differs. Regarding the technical units of ESVAC, this does not necessarily have an impact on the overall PCU. But—at least at the moment—the average antibiotics usage differs substantially between the species (Carmo et al., 2017; Obritzhauser et al., 2016; Postma et al., 2015; Schüpbach‐Regula, Torriani, Gassner, Stucki, & Müntener, 2009; Sjölund et al., 2016). Reasons for species differences are only partially due to the species itself—such as metabolism characteristics—but mainly result from different patterns regarding husbandry and type of use. Some animal species are kept under extensive conditions such as sheep and goats. Stocking densities are usually low and these species tend to have good health conditions as their breeding does not focus on high performance. Other species such as pigs and poultry are often farmed intensively, since their rapid growth allows for short production cycles. High stocking densities, fattening of young animals with insufficient immune system can cause high infection rates in intensive farming systems. In consequence, the animals need to be treated more frequently. This highlights the special challenge of reducing antibiotics usage in intensive animal husbandry which includes all aspects of farm management, animal husbandry and genetics.

At the moment, countries with a large amount of intensive animal husbandry tend to have higher antibiotics usage than other countries although countries with low sales figures despite intensive animal husbandry exist (e.g. Denmark, the Netherlands). Some European countries have made large improvements in the last years and were able reduce their antibiotics usage substantially (European Medicines Agency, 2017). Comparisons between countries should, hence, be based on data stratified for animal species, age group and husbandry system. This will facilitate to identify countries or regions in which special efforts in reducing antibiotics usage are necessary. The challenges regarding comparisons between populations are discussed in detail by Collineau et al. (2017). While Collineau et al. (2017) provide a complete and balanced review, the intention of this paper is to highlight reasons for the differences between countries that could be prevented using nDDDAvet or nUDDAvet instead.

### 4.3 Substances

Sales data must be analysed separately for each substance or substance class due to two reasons. First, the doses differ significantly between substances, animal species as well as between different administration routes (examples given below). Second, the substances and substance classes differ concerning their impact on selection of resistant bacteria.

### 4.4 Dosage

The main variables of substance selection are animal species, pathogen species, affected tissue (due to pharmaceutical characteristics and the respective availability in tissues) and route of administration. The availability and the price of the pharmaceuticals also appear to be important as well as habit, ease of application and marketing strategies of products.

The dosage of colistin in terms of weight of active substance per kg bodyweight is 5.1 mg/kg for poultry, whereas tylosin has a dose of 81 mg/kg (European Medicines Agency, 2016, 2015). Consequently, treatment with tylosin uses much more mg/PCU than treatment with colistin.

Doses also depend on the animal species. For example, tylosin has recommended dosages of 81 mg active substance/kg in poultry, 12–13 mg/kg in pigs, and 13–41 mg/kg in cattle, depending on the administration route (European Medicines Agency, 2016). This also influences the amount of substance used and can cause a bias in comparisons between countries.

Bias can also occur as the recommendations in the Summary of Product Characteristics (SPCs) vary between different pharmaceutical products, although they contain the same substance and refer to the same species, administration route and indication. These circumstances not only affect the mg/PCU but also the nDDDvet. Solely the nUDDvet is not affected as it considers the used dose instead of a defined dose.

### 4.5 Classification of substance groups

The second reason for substance‐specific analyses is that the usage of antimicrobials also affects the selection and distribution of bacterial strains that acquired resistance against specific antimicrobial substances (resistant bacteria). Each usage of antibiotics stimulates the selection of resistant bacteria and, thus, potentially contributes to future treatment failures in veterinary or human medical care. The substance classes are categorized depending (1) on the importance of the substance in veterinary medicine (e.g. if alternatives are available), (2) on the zoonotic relevance of the infectious diseases and (3) on the risk of transmission of resistant bacteria to humans (European Medicines Agency, 2014). The Advisory Group on Integrated Surveillance of Antimicrobial Resistance (AGISAR) of the World Health Organization (WHO) has recommended the restricted use of substances that are either the only or one out of few substances effective against a severe disease or that are effective against zoonotic pathogens. AGISAR classified the substances into one of three categories. Third‐ and fourth‐generation cephalosporins, macrolides, quinolones, glycopeptides as well as polymyxins are categorized as ‘highest priority critically important drugs’ (WHO Advisory Group on Integrated Surveillance of Antimicrobial Resistance (AGISAR) 2017).

As some critically important substance classes such as cephalosporins and fluoroquinolones have low doses, the usage of these instead of other substance classes can cause an overall sales reduction while increasing the risk for public health at the same time.

### 4.6 Quantification of defined treatments nDDDvet

Although defined doses are determined following the recommendations in the respective SPCs (e.g. European Medicines Agency, 2016) or expert opinions, they have to be regarded as a technical unit only. They do not necessarily reflect the most frequently used dose of the substance, since the used dose also depends on the veterinarian's personal decision. With regard to interpretation, the defined doses should be chosen as closely as possible to the used doses. Scientific studies such as Joosten et al. (2019) using several approaches of calculating the numbers of used or defined daily doses in a multi‐country study on broiler farms revealed differences within and between countries. The same research consortium ‘Ecology from Farm to Fork Of microbial drug Resistance and Transmission’ EFFORT yielded similar results in pig farms (Sarrazin et al., 2019).

The calculation of defined treatments allows for comparison of treatment frequencies between substances. It is also possible to look for trends and differences between countries. For assessment of antibiotics usage in animals concerning public health aspects, possible shifts from undesired substance classes such as fluoroquinolones or third‐/fourth‐generation cephalosporins to less critical substance classes can be observed.

In conclusion, the number of defined treatments is well suited for population or time period comparison. The main challenge is to point out that although this form of data presentation may seem to reflect the number of actual substance application to animals, it must be regarded as technical unit which is not identical to the actual number of treatments. Thus, it is recommended not to be used in communication with farmers. Nevertheless, in some countries such as Denmark and the Netherlands, this method is successfully employed for benchmarking of farms (Bager et al., 2015; SDa expert panel, 2016).

### 4.7 Quantification of actual treatments nUDDvet

The other approach is to determine the actual number of used daily doses, nUDDvet. The relevant information can only be collected on farm level and thus requires much effort (Menéndez González et al., 2010; Persoons et al., 2012; van Rennings et al., 2015).

Furthermore, data often originate from different sources, for example, farmers or veterinarians, and thus, reliability of data might be violated due to reporting bias. The possible (negative) consequences further decrease the willingness of data owners to submit data.

However, the nUDDvet is well suited for analysis and communication of antibiotic usage on farm or veterinarian level, as its calculation is based on real treatments and can be easily understood. Changes of treatment frequency within a farm can be displayed transparently and facilitates a more direct observation by the animal owner and the veterinarian, for example, of the success of measures taken to increase animal health. Regardless of the large time expenditure and effort, Germany successfully introduced a monitoring system in farm animals in 2014 (Bundesministerium für Ernährung & Landwirtschaft und Verbraucherschutz, 2^2013^; Bundestag, 2013).

### 4.8 Comparison of the different approaches

The results of the three calculation methods differ. If the substance is unknown, it is not possible to infer treatment frequency from the amount of substance used. Differences between nDDDvet and nUDDvet rely on differences between defined and actual dose or on differences between average and actual animal weight. Discrepancies between used and defined doses do not imply that an inappropriate dose was chosen, as appropriate dosing depends on certain conditions within the animal group such as age, health status or resistance situation. The discussion of this paper focuses on the possible differences in the assessment of the antibiotics treatment depending on the calculation approach. For a complete review, see Collineau et al. (2017).

Under practical conditions, decisions of veterinarians to treat animal flocks take different perspectives into account, the complexity of which is illustrated by the example of treating respiratory diseases such as Mycoplasma infection in broilers given above: the administration of tylosin uses the highest amount of substance, while treatment with the combination formula Lincomycin + Spectinomycin yields higher values of nDDDvet and nUDDvet. The responsible veterinarian is aware of these facts, but he or she needs to consider the results of microbiological investigations, severity of flock infestation including animal welfare and also consumer protection. All antimicrobial substance classes including those mentioned in the example given above need to be classified as critical considering both veterinary and human medicine. The WHO has classified macrolides ‘highest priority critically important antimicrobials’ and EMA recommends a restrictive use of this substance class due to possible selection for macrolide‐resistant Campylobacter in animals (European Medicines Agency, 2017; WHO Advisory Group on Integrated Surveillance of Antimicrobial Resistance (AGISAR) 2017; ; ).

However, the European Union´s report on antimicrobial resistance of zoonotic and indicator bacteria from humans, animals and food in 2016 reflects a low overall resistance for *Campylobacter jejuni* isolated from broilers against the important macrolide erythromycin, that is, 1.3% in average for 24 Member States (European Food Safety Authority & European Centre for Disease Prevention and Control, 2018).

Lincosamides such as Lincomycin are classified ‘highly important’ and used to treat important bacteria such as Enterococcus, Staphylococcus (MRSA) in human intensive care units. Aminoglycosides are preliminary rated in category 2 because a risk assessment has not yet been carried out (European Medicines Agency, 2014). In general, combination preparations display a higher risk for selection of resistant bacteria and thus should be avoided following guidelines of prudent use (European Commission, 2015). In summary, decision‐making remains difficult for the practitioner.

In conclusion, the assessment of antibiotics usage in animals is not straightforward. Some measures seem to be more precise concerning the frequency in which an animal is treated with antimicrobials on average. As these measures require much time and effort, they are applicable for studies with a limited (representative) sample size and may include reporting bias. Thus, the results underlie uncertainty and bias.

As sales data can be made available as complete and unbiased datasets by many countries, they are currently the best choice for comparisons between countries (Grave et al., 2014). This approach facilitates sampling of respective data and thus encourages countries to implement a monitoring system. It represents the necessary first step which is ideally followed by more detailed monitoring and surveillance systems focusing on the reduction of antimicrobial use in general or of critically important antimicrobials in the context of public health. EMA already initiated data collection activities on species level enabling the calculation of nDDDvet for different animal species/category separately (European Medicines Agency, 2018). As explained above, this change in methods will contribute to data stratification for animal species/categories and will additionally control the effects of substances.

Recently, several reviews have been published that discuss the methods available and their applications seriously. Interested readers may refer to Collineau et al. (2017) and to Werner et al. (2018).

Regardless of the method of data collection, monitoring of the antibiotics usage must be regarded as a tool to observe changes in the pattern of antibiotics use. The main goal is to reduce the risk for public health which requires the distinction between substances. Monitoring and surveillance should not only focus on a general reduction of antibiotics use. The risk of selection and distribution of resistant bacteria should also be minimized while protecting animal health and animal welfare at the same time. It is the task of veterinarians, animal owners, scientists as well as authorities to develop applicable approaches to enhance animal health and thus reduce the need of antimicrobials. The quantification of antibiotics usage in this context is only a tool and should not be misinterpreted as objective. Actions should focus on prudent use to reduce the burden of resistant bacteria and thus enhance not only public health but also animal health and welfare.

## CONFLICT OF INTEREST

All authors do not declare any competing interests and agree to the submission of the manuscript.

## ETHICAL STATEMENT

The authors confirm that the ethical policies of the journal, as noted on the journal's author guidelines page, have been adhered to. No ethical approval was required as this is an article with no original research data.
